# Bacillus velezensis UTB96 Is an Antifungal Soil Isolate with a Reduced Genome Size Compared to That of Bacillus velezensis FZB42

**DOI:** 10.1128/MRA.00667-19

**Published:** 2019-09-19

**Authors:** Maliheh Vahidinasab, Masoud Ahmadzadeh, Marius Henkel, Rudolf Hausmann, Kambiz Morabbi Heravi

**Affiliations:** a Institute of Food Science and Biotechnology, Department of Bioprocess Engineering (150k), University of Hohenheim, Stuttgart, Germany; b Department of Plant Protection, College of Agriculture and Natural Resources, University of Tehran, Karaj, Iran; Georgia Institute of Technology

## Abstract

Bacillus velezensis UTB96 was isolated from soil based on its antifungal activity. Whole-genome sequencing of strain UTB96 provided further information about its secondary metabolite gene clusters. Compared to the well-known strain FZB42, UTB96 lacks an IS*3* element and a type I restriction endonuclease.

## ANNOUNCEMENT

Bacterial strain Bacillus velezensis UTB96 was isolated from the soil of the pistachio tree in an extensive screening of the plant probiotic bacterial community in Kerman, Iran (1). Out of thousands of isolated strains, UTB96 had the highest antifungal activity against Aspergillus flavus. Likewise, strain UTB96 was able to degrade the aflatoxin produced by Aspergillus flavus on pistachio nuts (1, 2). Also, UTB96 showed antagonistic activity against important soilborne fungal phytopathogens, for example, Fusarium graminearum (M. Ahmadzadeh, unpublished data) or Phytophthora drechsleri (3). Such antifungal activity could be a result of the production of three lipopeptide families (surfactin, iturin, and fengycin) by strain UTB96 (1, 2, 4).

The previous identification of strain UTB96 was performed using biochemical tests and 16S rRNA gene sequencing (NCBI accession number KY992857), which showed that UTB96 is Bacillus amyloliquefaciens (2). To characterize the genetic background and identify the genes responsible for biosynthesis of lipopeptide and antifungal metabolites, whole-genome sequencing of strain UTB96 was performed. In detail, a single colony of UTB96 was used to inoculate LB medium, which was then incubated overnight at 37°C with shaking at 120 rpm. Afterwards, chromosomal DNA was extracted using an innuPrep bacterial DNA kit (Analytik Jena, Jena, Germany). The library preparation and whole-genome sequencing were carried out by Eurofins Genomics (Ebersberg, Germany). Genome sequencing was obtained using an Illumina HiSeq 4000 instrument and library prepared with the 2 × 150-bp paired-end read length, including DNA fragmentation, adapter ligation, amplification, and size selection. The quality of the final library was assessed by determination of size distribution and quantification. All of the steps were performed according to Eurofins Genomics protocols, resulting in a total 3,270,666 reads. Raw data analysis and sequencing assembly were carried out using Geneious software ver. 11.1.5 (Biomatters Ltd.). Unless otherwise specified, default software settings were used for all analyses. In a preliminary approach, after pairing the reads, the quality of paired-end reads was controlled by quality trimming. The adapter sequences and the sequences with low quality were trimmed with a quality limit of 0.05. The paired-end reads were merged, and chimeric reads were removed. DNA assembly was performed by *de novo* assembly. A total of 24 contigs were generated, including a 981,939-bp contig as the largest contig and an *N*_50_ contig size of 496,461 bp. A nucleotide BLAST search of this contig showed the highest similarity to *B. velezensis*. Based on this result and together with the nucleotide BLAST search of DNA topoisomerase-encoding genes, it was clear that UTB96 is a *B. velezensis* strain. Therefore, in the final and main approach, the genome sequence of strain *B. velezensis* FZB42 (GenBank accession number NC_009725) was used as a reference map for genome assembly of UTB96 using the raw sequencing data files. Comparative BLAST analysis of UTB96 by NCBI indicated its similarity to strain FZB42 (query coverage, 99%; identity, ≥97.99%).

The genome of strain UTB96 consists of a circular sequence of a 3,715,675-bp chromosome with a G+C content of 46.9%. *B. velezensis* strains are known to produce various secondary antimicrobial metabolites, such as lipopeptides and polyketides (5–7). Due to production of these metabolites, *B. velezensis* has the ability to exert an antimicrobial effect against many phytopathogenic microorganisms (8–10). Production of secondary metabolites by UTB96 was analyzed using the antiSMASH tool with the default setting (11). Genomic clusters responsible for the biosynthesis of antimicrobial secondary metabolites were observed. These clusters involve genes encoding lipopeptides of surfactin, bacillomycin D, fengycin, and bacillibactin and polyketides of bacillaene, difficidin, and macrolactin. Likewise, *B. velezensis* is considered a plant growth-promoting bacterium (PGPB) and is a promising candidate for replacing chemicals in sustainable agriculture and biofarming systems (12, 13). Unlike that of *B. velezensis* FZB42, the genome of strain UTB96 did not have the IS*3* element or a type I restriction endonuclease.

### Data availability.

The complete genome sequence of *B. velezensis* UTB96 has been deposited in the NCBI database under the GenBank accession number CP036527. The raw read data are available at the NCBI Sequence Read Archive under SRA accession number SRX5559694.
